# Theoretical, Equilibrium, Kinetics and Thermodynamic Investigations of Methylene Blue Adsorption onto Lignite Coal

**DOI:** 10.3390/molecules27061856

**Published:** 2022-03-12

**Authors:** Naim Hasani, Teuta Selimi, Altin Mele, Veprim Thaçi, Jeton Halili, Avni Berisha, Makfire Sadiku

**Affiliations:** 1 Department of Hydrotechnics, Faculty Civil Engineering, University of Prishtina, 10000 Prishtina, Kosovo; naim.hasani@uni-pr.edu; 2 Department of Chemistry, Faculty of Natural and Mathematics Science, University of Prishtina, 10000 Prishtina, Kosovo; teutaselimi3@gmail.com (T.S.); veprim.thaci@uni-pr.edu (V.T.); jeton.halili@uni-pr.edu (J.H.); avni.berisha@uni-pr.edu (A.B.); 3 Department of Chemistry, Faculty of Natural Science, University of Tirana, 1000 Tirana, Albania; altin.mele@fshn.edu.al

**Keywords:** methylene blue, coal, adsorption, equilibrium, isotherms, Monte Carlo, kinetic models, thermodynamic

## Abstract

The interaction of methylene blue (MB) dye with natural coal (collected from coal landfills of the Kosovo Energy Corporation) in aqueous solutions was studied using adsorption, kinetics, and thermodynamic data, and Monte Carlo (MC) calculations. In a batch procedure, the effects of contact duration, initial MB concentration, pH, and solution temperature on the adsorption process were examined. The Langmuir, Freundlich, Temkin, and Dubinin–Radushkevich (D–R) isotherms were used to examine the equilibrium adsorption data. The equilibrium data fit well to the Freundlich and Langmuir adsorption isotherm models; however, the Freundlich model suited the adsorption data to a slightly better extent than the Langmuir model. The kinetics experimental data was fitted using pseudo-first-order, first-order, pseudo-second-order, second-order, Elvoich equation, and diffusion models. The pseudo-second-order rate model manifested a superlative fit to the experimental data, while the adsorption of MB onto coal is regulated by both liquid film and intraparticle diffusions at the same time. Thermodynamic parameters, such as Gibbs free energy (Δ*G*^0^), enthalpy (Δ*H*^0^), and entropy (Δ*S*^0^) were calculated. The adsorption of MB was confirmed to be spontaneous and endothermic. The theoretical results were in agreement with the experimental ones.

## 1. Introduction

Concern over environmental preservation has grown throughout the years from a worldwide perspective. In recent decades, the exponential population growth and social civilization growth have been accompanied by changes in productivity and consumption habits, increasingly affluent lifestyles, resource use, and the ongoing development of industrial technologies, all of which have resulted in the rapid generation of municipal and industrial solid wastes, resulting in the world’s most intractable paradox [1,2,3,4,5]. The rapid rate of industrialization has resulted in a variety of difficulties, including water contamination, which is regarded to be one of the most dangerous problems. Industrial activities dump massive volumes of untreated wastewater into the environment on a regular basis causing harm to aquatic, plant, and human life [6]. Excessive discharge of inorganic/organic contaminants into water as a result of industrialization, agricultural activities, and urbanization has created a major environmental concern all over the world. For example, more than thousand different types of dyes are commercially available and used extensively in the leather, paper, plastics, tannery, cosmetics, rubber, paint, pharmaceutical, culinary, photography, and textile sectors to color their products, and these are some of the sources of dye-containing effluents [7]. The textile dyeing business consumes a considerable amount of pure water, putting it at the forefront of pollutants [8]. Annually, more than 7 × 10^5^ tons of dyestuffs are generated, with a considerable portion being released directly into aqueous effluents.

The presence of colored effluents in an aquatic habitat inhibits sunlight from reaching the benthic species, hence impeding photosynthesis [9]. Methylene blue (MB) is a cationic dye that is often used for coloring but is also employed in microbiology, surgery, and diagnostics. Though MB is not extremely dangerous, it can have some negative consequences.

In humans, acute MB exposure can result in accelerated heart rate, shock, Heinz body formation, cyanosis, jaundice, quadriplegia, and tissue necrosis. MB produces eye burns, which can result in lasting damage to the eyes of humans and animals.

Dyes, in general, are poorly biodegradable or resistant to environmental conditions and so present a major difficulty in the treatment of dye-containing wastewater [7]. Because of the difficulties of treating such streams using traditional physical, chemical, physicochemical, and biological treatment approaches, dye removal from wastewater discharge is a difficult environmental challenge. For the treatment of dye-containing effluents, a variety of physical and chemical treatment procedures such as adsorption, coagulation, precipitation, filtration, electrodialysis, membrane separation, and oxidation have been utilized [10,11]. The adsorption procedure is one of the most successful and cost-effective ways for removing colors from aqueous solutions.

Various adsorbents including activated carbon, sugarcane dust, algae, red algae, macrofungus, green algae, lichen, saw dust, bottom ash, fly ash, de-oiled soya, maize cob, peat, iron humate, mixed sorbents, microbial biomass, activated slag, waste product from agriculture, bentonite, magnetic nanoparticle, and coal were previously used for the removal of color and trace elements from wastewater [12,13,14,15,16,17,18]. The natural and waste materials seem to be feasible alternatives for dye removal because of their economic and eco-friendly features, availability in abundance and low cost. Activated carbon has a high capacity for organic adsorption, but nonetheless is a costly material.

This study is the first to investigate and present a detailed explanation of the adsorption of MB on natural coal. Because Kosovo’s lignite coal has a high adsorption capacity for MB and it is abundant in Kosovo as a natural resource (Kosovo has the third largest lignite reserves in Europe and the fifth in the world) it opens new possibilities for the effective and efficient removal of pollutant molecules via the sorption process.

Natural (untreated) coal extracted from coal landfills of the Kosovo Energy Corporation, accordingly at the power plant “Kosova B” was utilized in this study as an adsorbent for the removal of MB from aqueous solutions at different temperatures. Monte Carlo calculations, and thermodynamic and kinetic parameters were also studied.

## 2. Results and Discussion

### 2.1. Effect of Contact Time and Initial MB Concentration

Contact time and dye concentration have a significant role in determining the rate of dye absorption, the time necessary for the adsorbent–adsorbate system to reach equilibrium, and the adsorbent’s adsorption capacity. These variables are also relevant in isotherm research involving adsorption processes.

**Figure 1:**
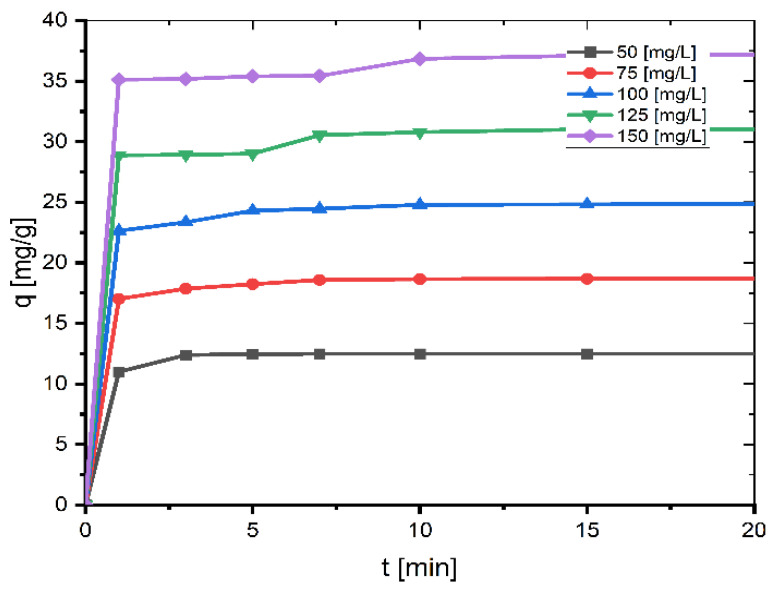
Effect of contact time and initial concentration on adsorption of MB onto coal (mass of coal = 0.1 g; volume of MB solution = 25 mL; *T* = 299.15 K, pH = 6.35).

The impact of agitation time and starting concentration on the adsorption of MB onto coal at 26 °C is depicted in Figure 1. The dye adsorption is rapid for the first ten minutes, the percentage of MB removed so far reaches a value of about 99% for small concentrations (50 mg/L, 75 mg/L, and 100 mg/L) and about 98% for larger concentrations (125 mg/L and 150 mg/L), and then slows as the surface of the coal becomes saturated with MB, reaching equilibrium after 60 min. When the contact period is 60 min, the greatest degree of absorption occurs. After increasing the agitation time further, the adsorption capacity stays nearly constant. Hence, the agitation duration was adjusted to 60 min in this investigation as this was the most appropriate contact time.

**Figure 2:**
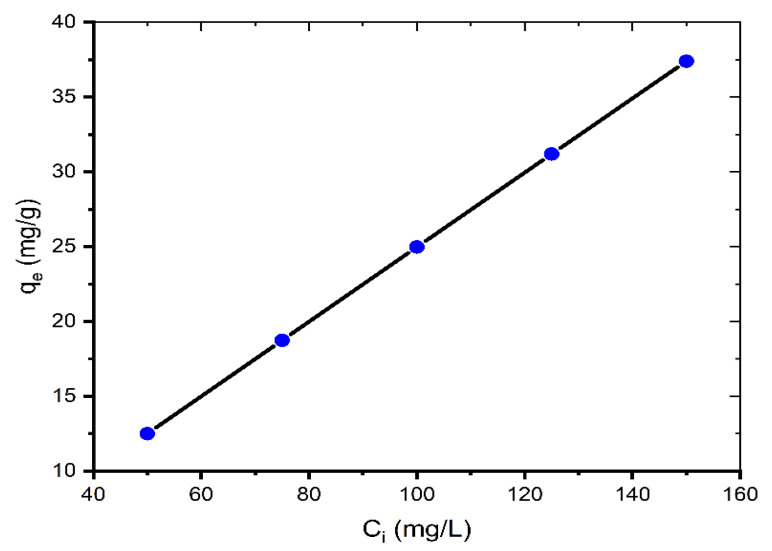
Effect of initial concentration on adsorption of MB onto coal (mass of coal = 0.1 g; volume of MB solution = 25 mL; *T* = 299.15 K, pH = 6.35).

Figure 2 represents the equilibrium absorption capacity versus the initial dye concentration (50–150 mg/L).

As the initial concentration increases, the adsorption capacity *q_e_* increases. The *q_e_* values are 12.50, 18.73, 24.97, 31.19, and 37.40 mg/g for 50, 75, 100, 125, and 150 mg/L, respectively. This is because when the initial concentration increases, the mass transfer driving force overcomes the resistances to dye molecule mass transfer from the solution to the solid phase, resulting in increased sorption. Additionally, increasing the concentration leads to greater contact between the dye molecule and the coal, which accelerates the sorption process [19]. Similar results for the contact time and initial MB concentration were also reported by other researchers [7,11,12,18,20].

### 2.2. Effect of pH

The influence of solution pH on the adsorption performance of the coal was investigated in the dye solutions (100 mg/L) of different pH (2.0–10.0).

**Figure 3:**
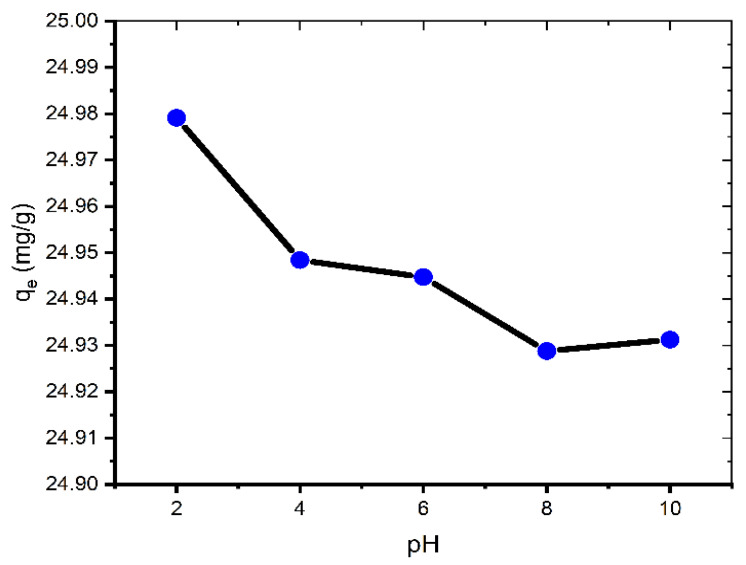
Effect of pH on adsorption of MB onto coal (contact time = 60 min; *C*_0_ = 100 mg L^−1^; mass of coal = 0.1 g; volume of MB solution = 25 mL; *T* = 299.15 K).

The adsorption of MB is very little affected by changing the pH of the solution (Figure 3). Therefore, we can say that pH value does not have any significant effect on MB adsorption onto coal and for that reason the experiments were conducted at ambient pH (pH = 6.35). This is an atypical case because most other studies exhibit a rise in the removal of MB with increasing pH [20].

### 2.3. Effect of Temperature and Thermodynamic Parameters

**Figure 4:**
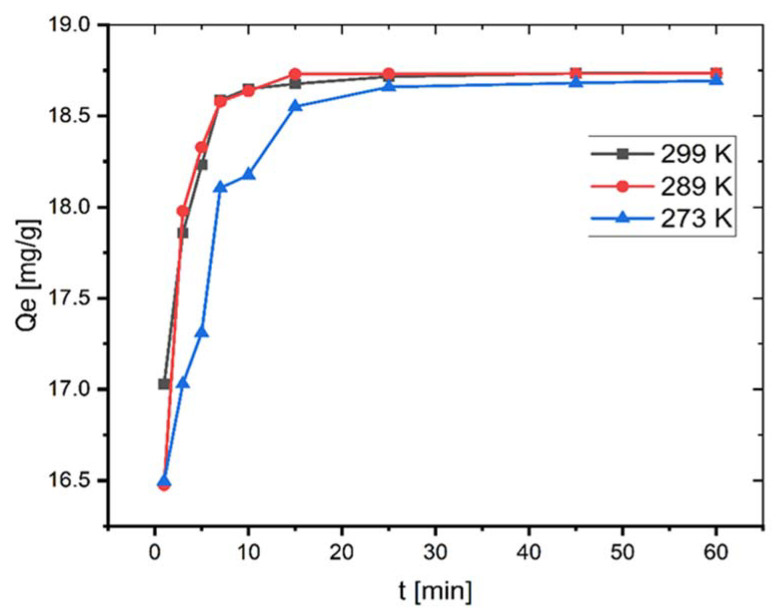
Effect of temperature on adsorption of MB onto coal (*C*_0_ = 100 mg L^−1^; mass of coal = 0.1 g; volume of MB solution = 25 mL; pH = 6.35).

The adsorption investigations were performed at different temperatures. As seen in Figure 4, *q_e_* increases, albeit only slightly, from 18.69 to 18.73 mg/g by increasing the temperature from 273–299 K.

**Figure 5:**
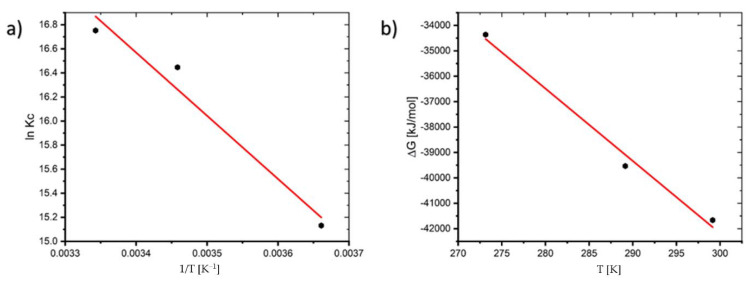
Plot of: (**a**) *lnK_c_* versus 1/*T* and (**b**) Δ*G* versus *T* of the adsorption process.

Plotting the linear plot of *lnK_c_* against 1/*T* from Equation (7), the thermodynamic variables Δ*H*^0^ and Δ*S*^0^ can be calculated from the slope and intercept, respectively (See Figure 5).

The thermodynamic variables at various temperatures are shown in Table 1. The negative value of Δ*G*^0^ indicates that the process is feasible and the value of Δ*G*^0^ decreases as the temperature increases, indicating that the process is more spontaneous at higher temperatures. The fact that Δ*H*^0^ (43.645 kJ/mol) is positive suggests that the reaction is endothermic. Adsorption is often classified as physical sorption when the Δ*H*^0^ value is smaller than 84 kJ/mol and chemisorption when the value is between 84 and 420 kJ/mol. Δ*S*^0^ is 286.14 J/mol∙K in this study, where the positive value shows that the disorder of the solid–liquid interface increases throughout the sorption process [20].

#### Activation Energy

**Figure 6:**
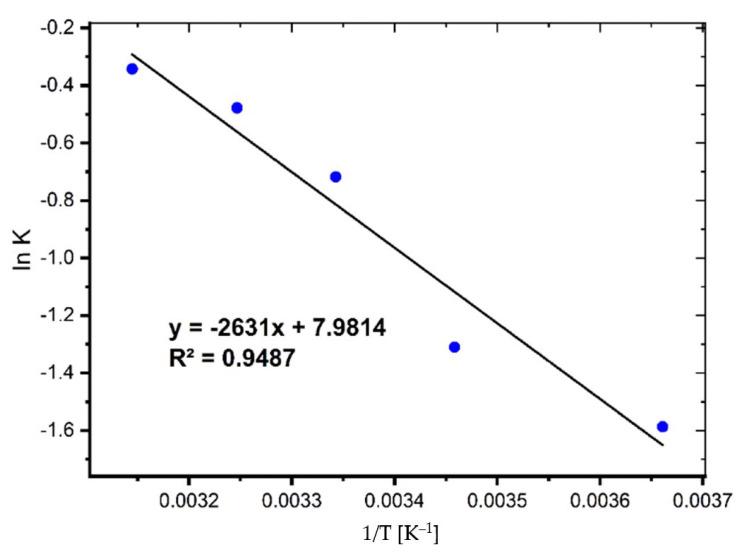
Activation energy plot for the adsorption of MB on coal.

The activation energy (*E_a_*) is a critical metric that indicates the strength and nature of the interactions that exist between MB and coal. The following linear Arrhenius equation was used to determine it [6]:

(1) $$lnk_{2}=lnA-\frac{E_{a}}{RT}$$

where *K*_2_ is the pseudo-second-order rate constant, *A* is the Arrhenius constant, *E_a_* refers to the energy of activation (J mol^−1^), *R* is the ideal gas constant (8.314 J∙mol^−1^∙K^−1^), and *T* is the temperature (K). The activation energy and Arrhenius constant were calculated using the slope and intercept of Equation (1) (See Figure 6).

The *E_a_* value was found to be 21.87 kJ/mol, showing that relatively modest forces are involved in the sorption process.

### 2.4. Adsorption Isotherms

**Figure 7:**
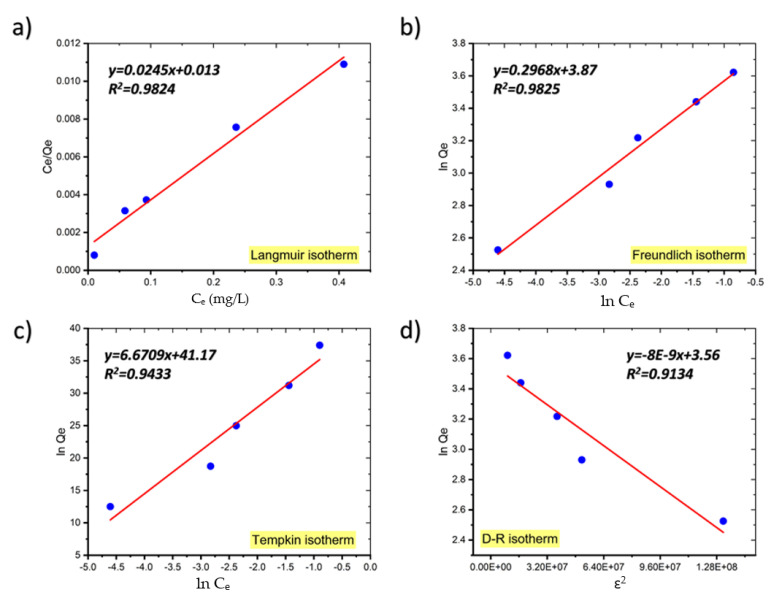
The Langmuir (**a**), Freundlich (**b**), Temkin (**c**), and Dubinin–Radushkevich (**d**) adsorption isotherm plots for the adsorption of MB onto coal.

The linear forms of the Langmuir, Freundlich, Temkin, and D–R isotherms for the adsorption of MB onto coal are shown in Figure 7 and Table 2 gives the parameters for each isotherm determined from the plot’s slopes and intercepts [21]. The isotherm with the best fit was chosen based on the highest correlation coefficient (*R*^2^) and the lowest value of the root mean square error (RMSE), which quantifies the isotherm’s fitness to the experimental data.

As shown in Table 2, although the equilibrium data fit well to both the Langmuir and Freundlich adsorption isotherm models, the Freundlich model fits the adsorption data just slightly better than the Langmuir model and the RMSE values for the Freundlich model are also smaller than those for the Langmuir model, indicating that the Freundlich isotherm model fits the experimental data better.

In general, if *n* > 1 it means that an adsorbate is favorably adsorbed on an adsorbent. The fact that in our study n is much greater than unity at all temperatures investigated indicates that coal is an appropriate adsorbent for MB adsorption from aqueous solution [21]. Similar findings have been published on the adsorption of crystal violet dye on coconut husk-based activated carbon, sorption of Acid Blue 161 by defatted microalgal biomass, and the removal of Reactive Black 5 dye by macadamia seed [22,23,24].

The adsorption process is considered to be favorable if the Langmuir isotherm’s *R_L_* value is between 0 and 1, i.e., 0 < *R_L_* < 1, linear when *R_L_* = 1, irreversible when *R_L_* = 0, and unfavorable when *R_L_* > 1. In our study, the values of *R_L_*, a critical parameter of the Langmuir isotherm, are between 0 and 1, indicating that the sorption process is favorable. Table 3 presents comparison of the maximum uptake of MB onto coal with that of various adsorbents.

Due to the low values of *R*^2^, the Temkin model does not reflect the data for the equilibrium isotherms of MB onto coal well [33].

Additionally, the Dubinin–Radushkevich (D–R) model was utilized to compute the apparent free energy of adsorption (E), which is often used to discriminate between physical and chemical adsorption [34]. The adsorption energy obtained from the D–R isotherms for the adsorption of MB on coal was below 8 kJ/mol (7.9 kJ/mol at 299 K, 7.07 kJ/mol at 289 K, and 4.08 kJ/mol at 273.15 K), suggesting that the uptake of MB onto coal was physical in its nature.

### 2.5. Kinetics

**Figure 8:**
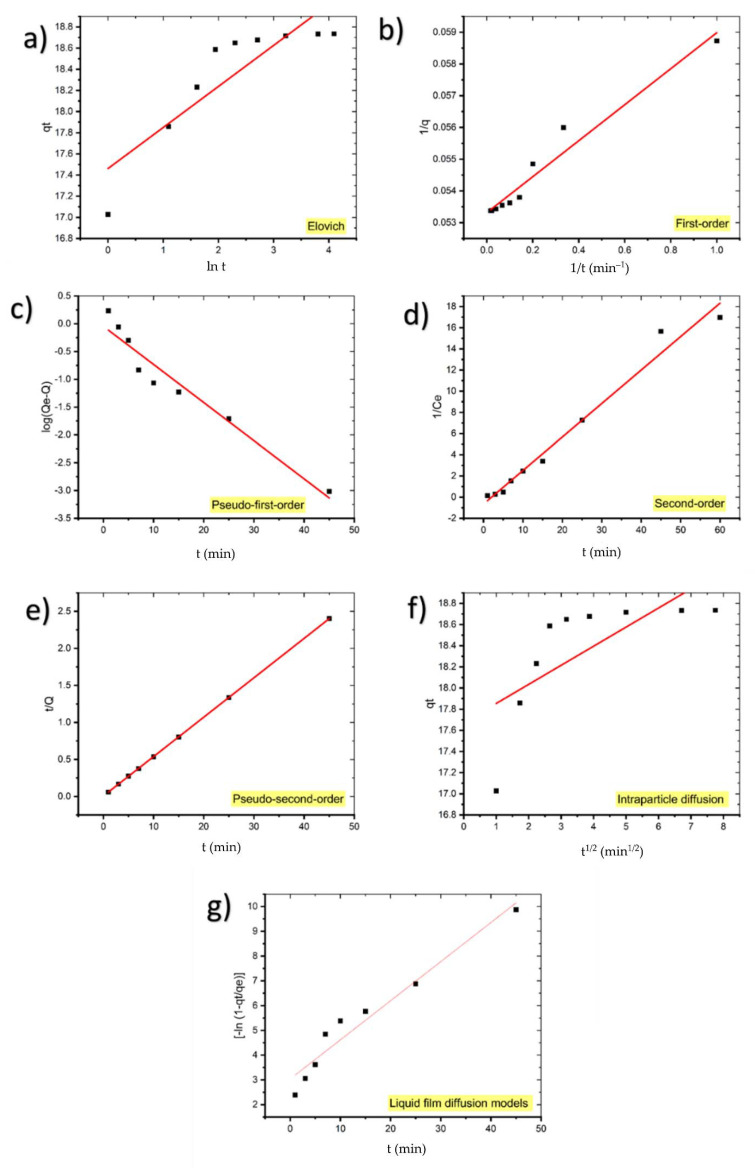
Kinetic models for the adsorption of MB onto coal: (**a**) Elovich, (**b**) first-order, (**c**) pseudo-first-order, (**d**) second-order, (**e**) pseudo-second-order, (**f**) intraparticle diffusion, and (**g**) liquid film diffusion models.

Table 4 summarizes the findings of fitting the experimental data to first-order, pseudo-first-order, second-order, pseudo-second-order, and diffusion models for MB adsorption onto coal. The linear relationship of kinetic models for the MB adsorption process using coal were shown in Figure 8.

The low value of the correlation coefficient of the Elovich model plot (*R*^2^ = 0.7755) indicates that the experimental data does not fit this model; hence it is not valid for this system.

The calculated *q_e_* value of pseudo-first-order kinetic (0.8993 mg/g) deviated from the experimental *q_e_* value (18.7353 mg/g) and *R*^2^ = 0.9454 suggests that the pseudo-first-order kinetic model does not fit well with the experimental results.

Although *q_e_* calculated for the first-order kinetic model (18.8679 mg/g) corresponds to the experimental value, the low value of *R*^2^ compared to the pseudo-second-order model shows that it is not the appropriate model for the adsorption of MB on coal.

Additionally, due to the low value of *R*^2^ (0.9796), the adsorption kinetics of MB onto coal do not obey the second-order kinetic.

As can be observed, the pseudo-second-order equation suited the data better than the other equations with *R*^2^ = 1. Additionally, the computed *q_e_* values from the pseudo-second-order model are highly consistent with the experimental results. These findings suggest that the adsorption system under consideration follows a pseudo-second-order kinetic model. Therefore, these results demonstrate that the adsorption magnitude may be due to the higher driving force causing fast transfer of MB molecules to the surface of the adsorbent particles and the availability of the uncovered surface area and the remaining active sites on the adsorbent [35]. Table 5 presents similar results achieved in other adsorption systems.

#### Diffusion Models

Due to the fact that surface diffusion, intraparticle diffusion, and adsorption are three phases in such systems, intraparticle and liquid film diffusion models were also used to determine the diffusion mechanisms.

It is expected that either liquid film diffusion, intraparticle diffusion, or both can act as rate-limiting steps [44]. If intraparticle diffusion is the mechanism underlying the adsorption process, the plot qt against *t*^1/2^ will be linear, and if the plot passes through the origin, the rate-limiting process will be solely due to intraparticle diffusion [45]. Otherwise, some additional process is involved in addition to intraparticle diffusion [46].

Because the intraparticle diffusion model plot has a low regression coefficient (0.5065) and the intercept is not equal to zero, it is unlikely that intraparticle diffusion was the only rate-limiting step; hence, kinetics was regulated by both liquid film and intraparticle diffusion concurrently.

### 2.6. MC Calculations

Distinguishing the best adsorption arrangement of the adsorbate molecules (MB) on the lignite surface is crucial for estimating the varied energy outputs. Calculation of the adsorption energetics of this technique is achievable by considering the interaction of the adsorbate molecules with the coal surface. This is performed quantitatively by finding the adsorption energy using the equation below:

(2) $$E_{adsorption}=E_{Lignite/MB}-\left(E_{Lignite\;}+E_{MB}\right)$$

where *E_Lignite/MB_* is the total energy of the simulated adsorption system, *E_Lignite_* and *E_MB_* are the total energy of the adsorbent and adsorbate molecules, respectively [47,48].

**Figure 9:**
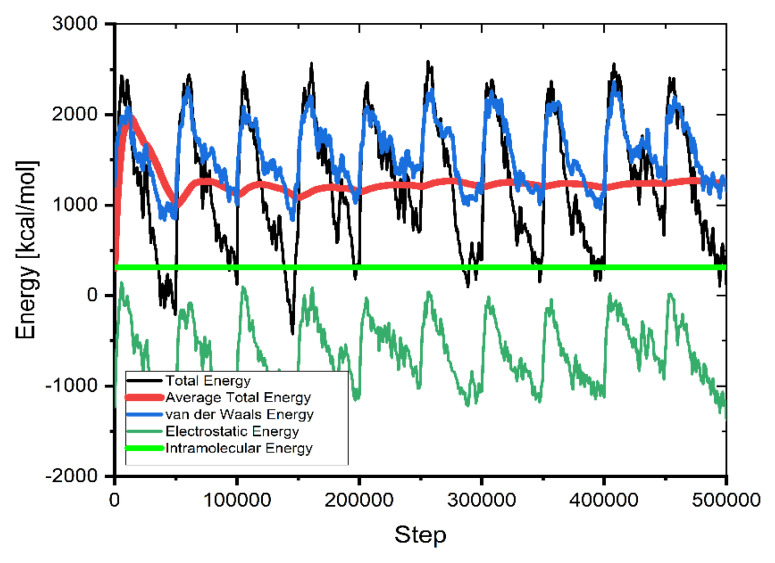
The contribution of the different energy terms during the MC calculations.

This method of calculating molecular interactions uses a large number of unsystematically generated types (molecules, ions) in the simulation box. As seen in Figure 9, the mean value of average energy flattens as supplementary configurations are tried, showing the system has attained energy equilibrium (after 3,000,000 steps).

**Figure 10:**
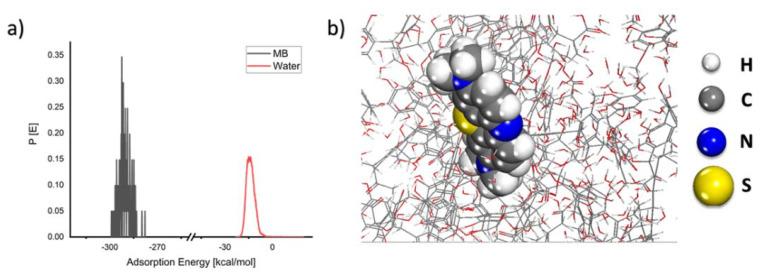
(**a**) Probability of the adsorption energy distributions during MC simulations of MB molecule adsorption onto the coal surface and (**b**) geometry of MB molecule adsorbed onto the coal surface as obtained from MC calculations.

As seen in Figure 10, the MB molecule forms an adsorption layer on the coal surfaces with a rather large negative energy value, indicating that the adsorption process is spontaneous. The theoretical results corroborate the experimental findings.

According to the adsorption energy determined from the MC calculations, lignite has a greater adsorption energy than clay minerals such as kaolinite [49]. Because lignite is a copious resource, it is an important material for exploration into the adsorptive removal of MB and other organic pollutants.

### 2.7. FTIR Spectroscopy

Total Attenuated Reflection (ATR) measurements were taken with an FTIR-8400S instrument and the following parameters: resolution of 2 cm^−1^, 100 scans, 500–3500 cm^−1^.

**Figure 11:**
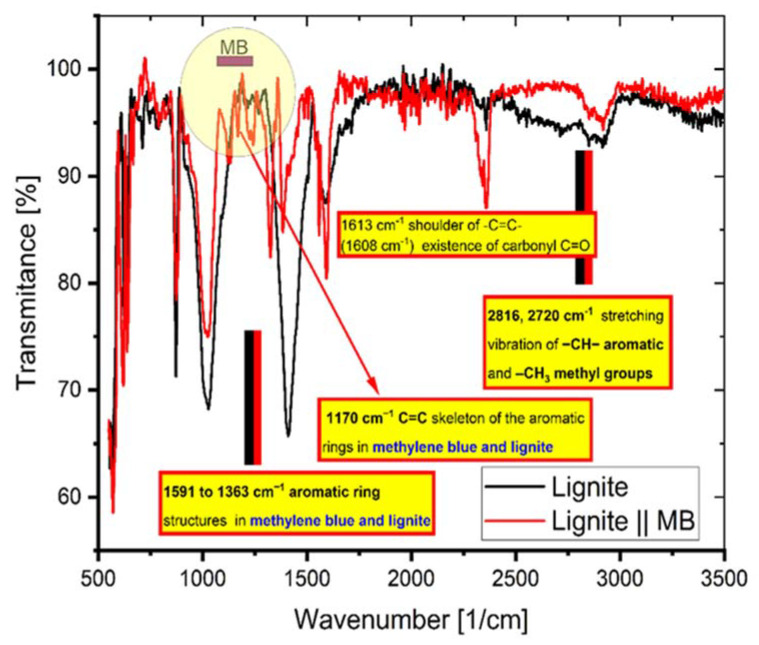
The FTIR spectrum of coal before and after adsorption of MB.

Figure 11 exhibits the peaks appearing at certain wave numbers of the coal FTIR spectra before and after the adsorption of MB. It indicates the presence of functional groups on the coal surface and that the adsorption of MB involves these groups.

The interaction of MB molecules with the functional groups of the coal was proved by the appearance and the vanishing or depletion of various peaks [18]. The following peaks: 2816 and 2720 cm^−1^, stretching vibration of –CH– aromatic and –CH_3_ methyl, respectively; 1591 to 1363 cm^−1^, aromatic ring vibrations; and 1170 cm^−1^, –C=C– vibrations from skeleton of the aromatic ring structures, arise from lignite and MB groups with distinctive features (as indicated in Figure 11).

## 3. Materials and Methods

### 3.1. Adsorbent and Adsorbate

Natural coal from the “Kosova B” power station was employed as an adsorbent in this investigation. The coal was ground first, and then dried and sieved via a 0.5 mm sieve. Without any chemical or physical activation, this material was employed directly for adsorption studies.

MB (basic blue 9, C.I. 52015; chemical formula: C_16_H_18_N_3_ClS; molecular weight: 319.85 gmol^−1^) supplied by Merck was employed as an adsorbate. One gram of MB dye was dissolved in one liter of distilled water to make a stock solution. The varied concentrations of working solutions were generated by diluting the stock solution with distilled water. Table 6 summarizes the dye’s properties and molecular structure [50].

### 3.2. Adsorption Experiments

Dye removal experiments were carried out in batch mode to explore the influence of various factors such as contact time (1–180 min), initial dye concentration (50, 75, 100, 125, and 150 mg/L), temperature (0, 16, and 26 °C), and pH (2–10) on removal of MB from aqueous solution. Batch tests were carried out by stirring 25 mL of known concentration MB solution with 0.1 g of coal using a magnetic stirrer.

The medium pH was regulated using solutions 0.1 M HCl and 0.1 M NaOH, respectively. After adsorption, the adsorbent and the supernatants were separated by centrifugation at 5000 rpm for 10 min and samples were examined for residual dye concentration using a UV–Visible Spectrophotometer (type T70+), with a 1 cm quartz cell. The amount of dye adsorption at equilibrium *q_e_* (mg/g) was calculated using Equation (3).

(3) $$q_{e}=\frac{\left(C_{i}-C_{e}\right)*V}{m},$$

where *C_i_* is the initial dye concentration (mg/L), *C_e_* is the equilibrium dye concentration (mg/L), *V* is the volume of MB solution used (L), and *m* is the mass of coal used (g).

The MB percent removal was calculated applying Equation (4).

(4) $$Removal\;\left(\%\right)=\frac{\left(C_{i}-C_{e}\right)*100}{C_{i}}$$

Origin 2019 b software was used to create all of the graphs. Regression analyses were also carried out by calculating *R*^2^ in order to examine the adequacy of a particular mathematical model. The RMSEs were calculated to assess the accuracy of the model predictions. The sum of the squares of the difference between dye removal experimental data (*q_exp_*) and model predictions (*q_ca__l_*) for each data set was divided by the number of data points (N), and the square root of this term was calculated as follows [6]:

(5) $$RMSE=\sqrt{\frac{\Sigma {\left(q_{exp-}q_{cal}\right)}^{2}}{N}}$$

By analyzing the sorption process at various temperatures, thermodynamic variables such as the Gibbs free energy, enthalpy, and entropy were explored (0, 16, and 26 °C). The change in Gibbs free energy is computed using *K_L_* from the Langmuir isotherm. The following variables were calculated using the van’t Hoff equation:

(6) $$\Delta G^{0}=-RT\;lnK_{c}$$

(7) $$lnK_{c}=\frac{\Delta S^{0}}{R}-\frac{\Delta H^{0}}{RT}$$

(8) $$K_{c}=K_{L}*10^{6}$$

where *k_c_* is the equilibrium constant (dimensionless) [51,52], *R* is the gas constant (J/Kmol), and *T* is the temperature (K).

### 3.3. Adsorption Isotherms

The Langmuir, Freundlich, Temkin, and Dubinin–Radushkevich isotherms were utilized in this work to explain the adsorption equilibrium and ascertain the validity of the experimental findings.

The Langmuir isotherm assumes monolayer adsorption onto a surface with a finite number of adsorption sites of uniform adsorption strategies and no adsorbate transmigration in the plane of the surface [53]. The nonlinear and linear versions of the Langmuir isotherm equation are as follows:

(9) $$q_{e}=\frac{q_{m}bC_{e}}{1+K_{L}C_{e}}$$

(10) $$\frac{C_{e}}{q_{e}}=\frac{1}{q_{m}K_{L}}+\frac{C_{e}}{q_{m}}$$

where *q_m_* is the monolayer adsorption capacity of the adsorbent (mg/g) and *K_L_* is the Langmuir adsorption constant (L/mg) which is related to the free energy of adsorption.

A plot of *C_e_*/*q_e_* against *C_e_* allows for the calculation of *q_m_* and *K_L_* from the slope and intercept of a straight line, respectively. The main properties of the Langmuir isotherm may be described by a dimensionless constant (the separation factor), *R_L_* [54].

It is given by the following equation:

(11) $$R_{L}=\frac{1}{1+K_{L}C_{0}}$$

where *C*_0_ (mg/L) is the highest initial dye concentration. *R_L_* specifies whether the isotherm is either unfavorable (*R_L_* > 1), linear (*R_L_* = 1), favorable (0 < *R_L_* < 1), or irreversible (*R_L_* = 0) [20].

The Freundlich model is an empirical equation that is based on sorption on heterogeneous surfaces or surfaces that support sites with varying affinities. The stronger binding sites are considered to be initially occupied and the binding strength diminishes with increasing site occupancy. Equation (12) expresses this empirical model.

(12) $$lnq_{e}=lnK_{F}+\frac{1}{n}lnC_{e}$$

where *K_F_* and *n* are Freundlich constants with *n* giving an indication of the favorability of the adsorption process, and *K_F_* (mg/g (L/mg)^1/*n*^) is the adsorption capacity of the adsorbent. *K_F_* is the adsorption or distribution coefficient and represents the amount of dye adsorbed onto the adsorbent at a unit equilibrium concentration [54].

The Temkin isotherm model accounts for the adsorption process’s indirect adsorbate/adsorbate interactions. Additionally, the model assumes that as the covering of the layer increases, the heat of adsorption of all molecules in the layer decreases linearly. Temkin’s linear form is as follows:

(13) $$q_{e}=\frac{RT}{b_{T}}lnK_{T}+\frac{RT}{b_{T}}lnC_{e}$$

where *R* is the common gas constant (8.314 J/mol K), *T* is the absolute temperature (K), *b_T_* is the Temkin constant related to the heat of sorption (J/mol) which indicates the adsorption potential (intensity) of the adsorbent, and *K_T_* (L/g) is the Temkin constant related to adsorption capacity.

The linear plots of *q_e_* versus *lnC_e_* enable the determination of *b_T_* and *K_T_* constants from the slope and intercept, respectively [55].

The Dubinin–Radushkevich (D–R) model is a more generic model that does not make any assumptions about a homogeneous surface or constant adsorption potential. Equation (14) expresses the D–R model [33], which provides information on the sorption process, whether it be chemisorption or physisorption.

(14) $$lnq_{e}=lnq_{m}-\beta \varepsilon^{2}$$

where *q_e_* is the amount of MB adsorbed per unit mass of adsorbent (mg g^–1^), *q_m_* is the maximum sorption capacity, and *β* is the activity coefficient related to the mean sorption energy *E* (kJ/mol); the latter can be computed using Equation (15) [19].

(15) $$E=\frac{1}{{\left(2\beta \right)}^{1/2}}$$

where *ε* is the Polanyi potential, which is expressed by Equation (16):

(16) $$\varepsilon =RTln\left(1+\frac{1}{C_{e}}\right)$$

where *R* is the gas constant (J mol^–1^ K^–1^) and *T* is the temperature (K). Additionally, *b* (mol^2^ J^2^) and *q*_0_ can be obtained from the slope and the intercept of the plot of *lnq_e_* against *ξ*^2^, respectively [21].

### 3.4. Adsorption Kinetics

Adsorption kinetic studies are necessary in the treatment of aqueous effluents because they give essential information on the mechanism of the adsorption processes [56]. The adsorption data were analyzed using a variety of available kinetic models. The experimental data from the contact time experiments were used in this case. The Elovich, first-order, pseudo-first-order, second-order, and pseudo-second-order kinetics are denoted by their linear forms:

(17) $$q_{t}=\frac{ln\left(a*b\right)}{b}+\frac{lnt}{b}$$

(18) $$\frac{1}{q_{t}}=\frac{1}{q_{e}}+\frac{k_{1}}{q_{e}t}$$

(19) $$ln\left(q_{e}-q_{t}\right)=ln\;q_{e}-k_{1}t$$

(20) $$\frac{1}{C_{e}}-\frac{1}{C_{0}}=k_{2}t$$

(21) $$\frac{t}{q_{t}}=\frac{1}{k_{2}q_{e}^{2}}+\frac{t}{q_{e}}$$

where *a* (mg g^−1^ min^−1^) gives the rate constant and *b* (g mg^−1^) gives the rate of adsorption at zero coverage in the Elovich model. Additionally, *k*_1_ (min^−1^) is the first-order rate constant, *q_e_* and *q_t_* are the amounts of MB adsorbed per gram of adsorbent (mg g^−1^) at equilibrium and at time *t*, respectively, and *k*_2_ (mg g^−1^ min^−1^) is the second-order rate constant [6].

From the respective plots (Figure 8), the parameters for the kinetic models were determined and are given in Table 4.

#### Diffusion Models

Diffusion models are based on a three-step adsorption process: (1) diffusion across the liquid film surrounding the adsorbent particles (film diffusion); (2) diffusion within the pores and/or along the pore walls, referred to as internal diffusion or intraparticle diffusion; and (3) adsorption and desorption between the adsorbate and the active sites, referred to as mass action [57]. During the transport of adsorbate molecules from the liquid phase to the solid phase in film diffusion the boundary plays a critical role. The liquid film diffusion model can be used in the form expressed in Equation (22).

(22) $$ln\left(1-F\right)=-K_{fd}t$$

where *F* = *q_t_*/*q_e_* is the fractional attainment of equilibrium. A linear plot of −*ln* (1 − *F*) against *t* with a zero intercept would suggest that the kinetics of the sorption process are controlled by diffusion through the liquid film surrounding the solid sorbent.

The Weber–Morris model is the most often utilized; see Equation (23). Weber and Morris discovered that in many circumstances of adsorption, solute uptake is almost proportional to *t*^1/2^ rather than to the contact duration *t* [58].

(23) $$q_{t}=K_{int}t^{1/2}$$

where *K_int_* is the intraparticle diffusion rate constant. A plot of *q_t_* versus *t*^1/2^ should be a straight line with a slope of *K_int_* when intraparticle diffusion is the rate-limiting step. The straight line should pass through the origin if intraparticle diffusion is the sole mechanism for adsorption.

### 3.5. Molecular Modelling and Monte Carlo (MC) Calculations

**Figure 12:**
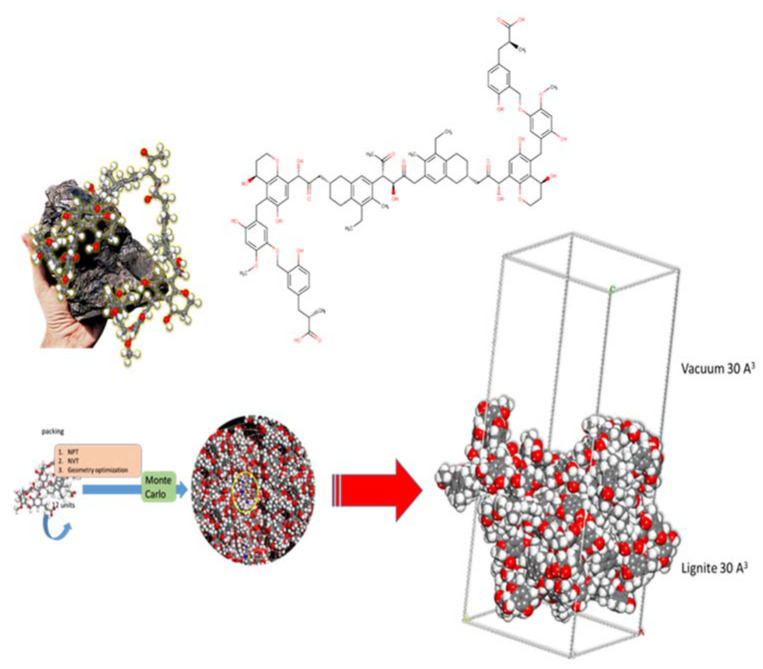
Representation of the parts of 3D and 2D chemical structures used as a starting point to construct Periodic Boundary Conditions (PBC) model of lignite used in Monte Carlo calculations (via packing, NPT, NVT, and geometry optimization).

The lignite model is based upon the literature (Figure 12). Monte Carlo calculations were completed (using the well-established Condensed-phase Optimized Molecular Potentials for Atomistic Simulation Studies—COMPASS II forcefield) under Periodic Boundary Conditions (PBC) using a lignite coal model (cell size of 30.222 Å × 30.222 Å × 30.222 Å) with a 30 Å vacuum layer encompassing: 1 MB molecule and 800 water molecules. The calculations gave molecular level information regarding the adsorption energy and geometry of the MB adsorption onto the studied lignite coal model.

The MC calculations were executed using 10 cycles of simulated annealing with 5,000,000 steps for each process. The temperature of the annealing process was set automatically from 105 to 102 K, for each cycle. Probable adsorption configurations were acquired as the temperature was gradually decreased.

The conclusions (the negative value of the MB adsorption energy, see Figure 8a) of the Monte Carlo simulations align well with the results of the experiments. The concept that the adsorption process is spontaneous is established by the fact that the adsorption energies are negative [59].

## 4. Conclusions

The current work demonstrates that natural coal may be employed as a very inexpensive adsorbent for the removal of MB from aqueous solution. It was found that the Freundlich model is a better fit for the adsorption of MB to natural coal than the other models considered. As expected, the MB adsorption process followed pseudo-second-order kinetics and the computed *q_e_* values correspond well with the actual values at given MB concentrations with high regression coefficients. An *E_a_* value of 32.19 kJmol^−1^ was determined showing that relatively modest forces are involved in the sorption process. Additionally, the mean free energy below 8 kJmol^−1^, derived from the D–R isotherm, and the Δ*H*^0^ value smaller than 84 kJ/mol demonstrate that the adsorption process is physically regulated.

The negative values of Δ*G*^0^ denote the spontaneous nature of adsorption, whereas the positive values of Δ*H*^0^ denote endothermic adsorption. The positive value of Δ*S*^0^ indicates that randomness is rising at the solid/liquid interface during MB adsorption on coal in aqueous solution. The thermodynamic analysis revealed that sorption is an endothermic, spontaneous, and physical process. MC calculations support the obtained experimental results.

## Figures and Tables

**Table 1:** The thermodynamic variables at different temperatures.

Temperature (K)	Δ*G*^0^ (kJ/mol)	Δ*H*^0^ (kJ/mol)	Δ*S*^0^ (J/mol∙K)
273.15	−41.66	43.645	286.14
289.15	−39.54		
299.15	−34.36		

**Table 2:** The data of equilibrium models studied.

Model	Equation	Parameters			
			299.15 K	289.15 K	273.15 K
Langmuir	$q_{e}=\frac{q_{m}bC_{e}}{1+bC_{e}}$	*q_m_* (mg g^−1^)	40.82	42.37	48.78
		*K_L_* (L mg^−1^)	18.85	13.88	3.73
		*R_L_*	0.0007	0.0005	0.0053
		*R* ^2^	0.9824	0.9897	0.9584
		RMSE	3.18	4.54	23.14
Freundlich	$q_{e}=K_{F}C_{e}{}^{1/n}$	*K_F_* (mg g^−1^)	48.13	51.41	41.37
		*n*	3.34	2.77	2.17
		*R* ^2^	0.9825	0.991	0.9620
		RMSE	2.12	0.87	1.91
Temkin	$q_{e}=\frac{RT}{b_{T}}ln(K_{T}C_{e})$	*K_T_* (L mg^−1^)	476.32	196.29	40.50
		*RT*/*b* (kJ/mol)	6.67	8.17	10.28
		*R* ^2^	0.9433	0.9814	0.9336
		RMSE	2.09	1.20	2.26
D–R	$q_{e}=q_{m}\exp\left(-\beta \varepsilon^{2}\right)$	*q_m_* (mg g^−1^)	35.35	38.48	36.23
		*β*	8 × 10^–9^	1 × 10^−8^	3 × 10^−8^
		*E* (kJ/mol)	7.9	7.07	4.082
		*R* ^2^	0.9134	0.9682	0.8935
		RMSE	2.99	1.80	3.08

**Table 3:** Comparison of the maximum uptake of MB onto coal with that of various adsorbents.

Adsorbent	*q*_max_/mg g^–1^	Ref.
Cedar cone	4.55	[25]
Fly ash	10	[26]
Modified coir pit	14.9	[27]
Microwave-treated nilotica leaf	24.39	[28]
Baker’s yeast	25	[29]
Natural coal	40.82	This study
Kaolinite	46.08	[30]
Sugarcane baggas	51.5	[31]
Jute stick powder	87.7	[32]
Acid-treated dika nut	232	[33]

**Table 4:** Kinetic parameters for the sorption of MB onto coal.

Model	Linear Equation	*q_e_*_exp_ (mg/g)	Parameters	
Elovich	$q_{t}=\frac{ln\left(a\;*\;b\right)}{b}+\frac{lnt}{b}$	18.7353	*a* (mg g^−1^ min^−1^)	1.34 × 10^19^
			𝑏 (gmg^−1^)	2.58
			*R* ^2^	0.7755
First-order	$\frac{1}{q_{t}}=\frac{1}{q_{e}}+\frac{k_{1}}{q_{e}t}$		*k*_1_ (min^−1^)	0.1418
			*q_e,calc_* (mg g^−1^)	18.8679
			*R* ^2^	0.9592
Pseudo-first-order	$ln\left(q_{e}-q_{t}\right)=lnq_{e}-k_{1}t$		*k*_1_ (min^−1^)	0.158
			*q_e,calc_* (mg g^−1^)	0.8993
			*R^2^*	0.9454
Second-order	$\frac{1}{C_{e}}-\frac{1}{C_{0}}=k_{2}t$		*k*_2_ (gmg^−1^ min^−1^)	0.3162
			*R^2^*	0.9796
Pseudo-second-order	$\frac{t}{q_{t}}=\frac{1}{k_{2}q_{e}{}^{2}}+\frac{t}{q_{e}}$		*k*_2_ (gmg^−1^ min^−1^)	0.488
			*q_e,calc_* (mg g^−1^)	18.797
			*R* ^2^	1
Intraparticle diffusion	$q_{t}=k_{i}\sqrt{t}+C$		*k_i_* (mg g^−1^ min^−1/2^)	0.1805
			*C*	17.673
			*R* ^2^	0.5065
Liquid film diffusion	$-ln\left(1-\frac{q_{t}}{q_{e}}\right)=k_{fd}t+C$		*K_fd_*	0.158
			*C*	3.037
			*R^2^*	0.9454

**Table 5:** Comparison of kinetic model for the adsorption of MB onto coal with other published works.

Adsorbent	Adsorbate	Kinetic	Ref.
Chitosan composite MCs/MS	RB19	Pseudo-second-order	[36]
Carbon of Quercus brantii (oak)	ACT	Pseudo-second-order	[37]
BS−HVL	MGC	Pseudo-second-order	[38]
α-Fe_2_O_3_@PHCMs	Methyl violet	Pseudo-second-order	[33]
Modified rice husk	Malachite green	Pseudo-second-order	[39]
Natural coal	MB	Pseudo-second-order	This study
Coal acid mine drainage	Fe	Pseudo-second-order	[40]
Charcoal (tree branches)	MB	Pseudo-second-order	[41]
Coal fly ash (CFA)	MG	Pseudo-second-order	[42]
Porous poly(imide-ether)s	MB	Pseudo-second-order	[43]

**Table 6:** Physical characteristics and molecular structure of Methylene blue.

Dye Name	Methylene Blue
Suggested name	Methylene blue
Abbreviation	MB
C.I. name	Basic blue 9
C.I. number	52015
Class	Thiazin
λ_max_	668 nm
Color	Blue

## Data Availability

Not applicable.

## Sample Availability

Samples of the compounds are available from the authors.
